# De-ubiquitination of ELK-1 by USP17 potentiates mitogenic gene expression and cell proliferation

**DOI:** 10.1093/nar/gkz166

**Published:** 2019-03-11

**Authors:** Charles Ducker, Leo Kam Yuen Chow, Janice Saxton, Jürgen Handwerger, Alexander McGregor, Thomas Strahl, Robert Layfield, Peter E Shaw

**Affiliations:** Transcription and Molecular Signalling Laboratory, School of Life Sciences, University of Nottingham, Queen’s Medical Centre, Nottingham NG7 2UH, UK

## Abstract

ELK-1 is a transcription factor involved in ERK-induced cellular proliferation. Here, we show that its transcriptional activity is modulated by ubiquitination at lysine 35 (K35). The level of ubiquitinated ELK-1 rises in mitogen-deprived cells and falls upon mitogen stimulation or oncogene expression. Ectopic expression of USP17, a cell cycle-dependent deubiquitinase, decreases ELK-1 ubiquitination and up-regulates ELK-1 target-genes with a concomitant increase in *cyclin D1* expression. In contrast, USP17 depletion attenuates ELK-1-dependent gene expression and slows cell proliferation. The reduced rate of proliferation upon USP17 depletion appears to be a direct effect of ELK-1 ubiquitination because it is rescued by an ELK-1(K35R) mutant refractory to ubiquitination. Overall, our results show that ubiquitination of ELK-1 at K35, and its reversal by USP17, are important mechanisms in the regulation of nuclear ERK signalling and cellular proliferation. Our findings will be relevant for tumours that exhibit elevated USP17 expression and suggest a new target for intervention.

## INTRODUCTION

The ETS transcription factor ELK-1 is acutely stimulated by mitogens to establish a gene expression programme commensurate with cell proliferation (1–4). ELK-1 is one of three ternary complex factors (TCFs) (5–7) and binds with serum response factor (SRF) to serum response elements (SREs) in a subset of target gene promoters. In the mouse, TCFs appear to be redundant (8–10), but this arrangement is not conserved because in other chordates depletion of a single TCF gene causes profound developmental defects (2,11–14).

Latent, nuclear ELK-1 acquires activity upon phosphorylation by MAPKs and deSUMOylation by PIASx (15–18). Target gene activation by phospho-ELK-1 involves recruitment of active ERK to chromatin (19), phosphorylation of mediator subunits including MED14 (20) and, uniquely for ELK-1 among the TCFs, functional reliance on MED23 (21,22). In proliferating human ES cells (hESCs), ELK-1 also locates to the promoters of differentiation genes independently of ERK and is associated with their repression (2).

Developmental regulators are subject to multiple levels of control. Mechanisms that attenuate key transcription factors include binding of specific repressor proteins (23), post-translational modifications (24), nuclear export (25), proteolytic cleavage (26) and proteasomal degradation (27). The ubiquitin–proteasome system (UPS) is known to influence levels of ELK-1, in particular the neuronal-specific isoform sELK, which is rapidly degraded in non-neuronal cells due to activation of a cryptic degron (28,29).

Here, we report a new mode of ELK-1 regulation involving its reversible ubiquitination. Mono-ubiquitin or low molecular weight ubiquitin species are conjugated to the ETS domain of ELK-1, predominantly to lysine 35 (K35). For convenience, we refer hereafter to these ELK-1-ubiquitin conjugates collectively as mono-ubiquitinated ELK-1 (ELK-mUBQ). In mitogen-stimulated or oncogene-expressing cells, i.e. when ERK activity is elevated, levels of ELK-mUBQ decline. We show that ELK-1 is a client of the ubiquitin-specific protease 17 (USP17) and that the two proteins interact directly. When expressed ectopically USP17 markedly decreases levels of ELK-mUBQ, augments ELK-1 activity and increases expression of ELK-1 target genes. Conversely, USP17 knockdown leads to the accumulation of ELK-mUBQ and attenuates ELK-1 transcriptional activity.

USP17 expression is cell-cycle regulated, but its elevated expression is a hallmark of multiple cancers and has been linked to aggressive tumour phenotypes (30–32). We find that USP17 depletion decreases cell proliferation and that expression of an ELK-1(K35R) mutant partially rescues this effect. Our data show that by reversing ELK-1 mono-ubiquitination USP17 augments transcriptional responses to ERK signalling that promote cell proliferation, events that are central to malignant cell growth.

## MATERIALS AND METHODS

### Cell culture and extract preparation

HEK293, HEK293T and HeLa cells were grown in Dulbecco’s MEM (low glucose) supplemented with 10% foetal calf serum (FCS), 2 mM l-glutamine, 100 U ml^−1^ penicillin and 100 μg ml^−1^ streptomycin. DU145 cells were grown in Dulbecco’s MEM (high glucose) with the same supplements. Whole cell extracts (WCEs) were prepared in a modified RIPA buffer; HeLa nuclear extracts were prepared as described earlier (19).

### Plasmids and DNA transfection

Plasmids and sources are listed in the Supplementary Experimental Procedures. Calcium phosphate/DNA co-precipitation was used to transfect HEK293/T cells, and PEI or LT1 (Mirus) was used to transfect HeLa cells.

### Ubiquitination assays

Cells were lysed in buffer containing 6 M guanidinium–HCl and protein–ubiquitin conjugates were captured by immobilized metal affinity chromatography (IMAC) on Nickel-Agarose beads (Qiagen) and washed in 8 M urea. After release from the beads conjugates were resolved by sodium dodecylsulphate-polyacrylamide gel electrophoresis (SDS-PAGE) and detected by immunoblotting. The antibodies used are listed in Supplementary Experimental Procedures.

*In vitro* ubiquitination assays were performed as described elsewhere (33). Briefly, ^35^S-labelled ELK-1 was generated by cell-free expression using the coupled TNT reticulocyte lysate system (Promega). After removal of unincorporated ^35^S-methionine by gel filtration (Micro-Spin, BioRad), radio-labelled ELK-1 was incubated with UBQ (10 μg), rE1 (500 ng), rE2 (UBCH5; 500 ng), ATP (4 mM), DTT (1 mM), ubiquitin aldehyde (5 μM) and HeLa Nxt (15–30 μg) for 1 h at 30°C. Reactions were resolved on 8% SDS-PAGE gels, dried and visualized by phosphor-imaging (Fujifilm).

### Protein mass spectrometry

HEK293T cells transfected with a vector encoding His-tagged ELK-1 were starved for 24 h or starved and stimulated by addition of 15% FCS and tetradecanoylphorbol acetate (TPA) (100 ng ml^−1^) before harvesting and protein enrichment by denaturing IMAC. On-bead samples were reduced (50 mM dithiothreitol), alkylated (100 mM chloroacetamide) and digested with sequencing-grade trypsin (0.02 mg/ml). They were then submitted to tandem mass spectrometry (MS/MS) on an LTQ-Orbitrap-Velos spectrometer with nano-flow liquid chromatography (LC). Identification of peptides was conducted in data-dependent mode. The raw data file obtained from each LC-MS/MS acquisition was run against the Uniprot human database. Data were analysed and di-gly modified peptides (+114) identified using Scaffold proteome software, combining Mascot and X! tandem search engines to validate assigned spectra.

### DNA-binding assays

Electrophoretic mobility shift assays (EMSAs) were performed as described previously (29). Southwestern assays were performed as described (34) with slight modifications.

### Gene expression assays

For reporter assays, cells in 24-well plates were lysed 36 h post transfection in passive lysis buffer; firefly and renilla luciferase expression were analysed with the Stop and Glow system (Promega). Quantitative reverse transcription-polymerase chain reaction (qRT-PCR) analyses were performed as previously described (20). Gene-specific primers and probes are listed in the Supplementary Data.

### Protein interaction assays

Co-immunoprecipitations were performed as described previously (20). GST-USP17 fusions expressed in bacteria were purified on glutathione agarose beads and incubated with recombinant ELK-1 or ERK2. Bound protein complexes were washed and resolved in SDS-PAGE for detection by immunoblotting.

### Intracellular protein distribution

Nuclear and cytosolic fractions of HEK293T cells transfected with vectors for ELK-1 and control shRNA or shUSP17#1 were isolated as described previously (29) and resolved in SDS-PAGE for protein detection by immunoblotting. For high content imaging, HeLa cells were transfected with vectors for ELK-1-GFP, ELK-1(R65)-GFP and control shRNA or shUSP17#2, fixed and stained with Hoechst H33342. Levels of ELK-1-GFP in nuclear and cytosolic compartments were determined using a Molecular Devices IX Ultra confocal plate reader and MetaXpress 6 software. Ratios of cytosolic/total GFP fluorescence were calculated for each GFP-positive cell and averaged for all cells in an ROI. Data points represent average ROI values (*n* = 48).

### Cell proliferation assays

One day post transfection HEK293T cells were trypsinized, counted and re-seeded into multiple 96-well plates at a density of 2 × 10^3^ cells per well. Cell proliferation was monitored daily using an MTT assay (35). Alternatively, transfected cells were re-seeded into 24-well plates at a density of 1 × 10^4^ cells per well and counted after 4 days using the Moxi Z Mini Automated Cell Counter (Orflo).

### Statistics

Reporter assay, qRT-PCR, cell counting and high content imaging data are expressed as mean ± SEM. Statistical analyses were performed with Student’s *t-*test. Proliferation (MTT) assays were analysed using ANOVA with Tukey’s multiple comparisons test. Significance is reported in figures by *P < 0.05, **P < 0.01, ***P < 0.001 and ****P < 0.0001.

## RESULTS

### ELK-1 is reversibly mono-ubiquitinated in proliferating cells

**Figure 1.**
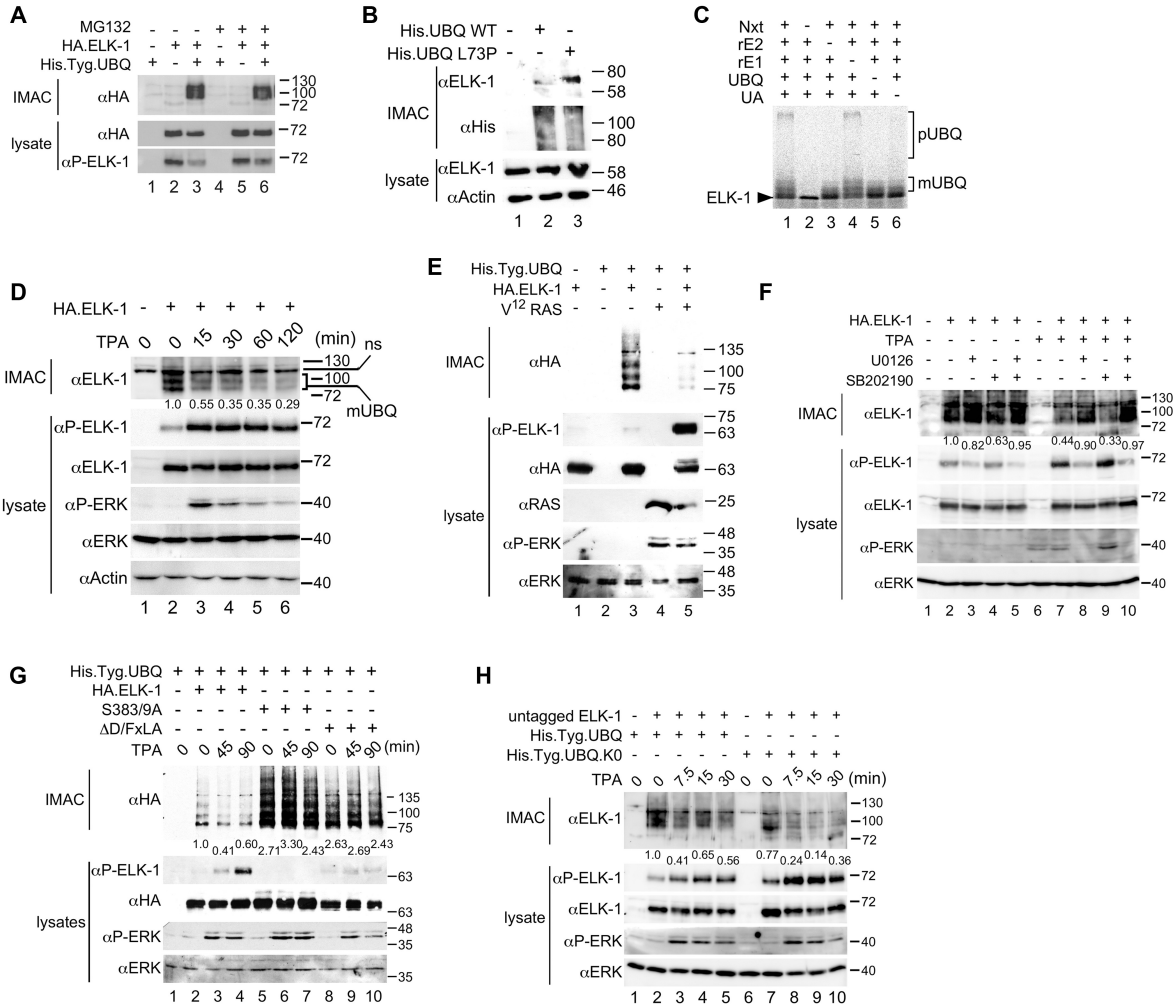
ELK-1 is reversibly mono-ubiquitinated. (**A**) WCEs from HEK293 cells transfected with expression vectors for His.Tyg.UBQ and HA.ELK-1 were subjected to IMAC. Where indicated cells were treated with MG132 (10 μM) for 8 h prior to harvest. Isolated proteins were analysed by SDS-PAGE (7.5%) and immunoblotting with the antibodies indicated. Lower panels: WCEs analysed for ELK-1 expression. (**B**) WCEs from HEK293T cells transfected with an expression vector for His.UBQ-WT or His.UBQ(L73P) were subjected to IMAC. Isolated proteins were analysed by SDS-PAGE (5–20%) and immunoblotting with the antibodies indicated. Lower panels: WCEs analysed for protein expression as indicated. (**C**) *In vitro* ubiquitination of ^35^S-labelled ELK-1 incubated with HeLa nuclear extract (Nxt) in the presence of recombinant E1, recombinant E2, UBQ and ubiquitin aldehyde (UA) as indicated. Reactions were separated by SDS-PAGE (7.5%) and analysed by phosphor-imaging. Brackets indicate poly-ubiquitylated (pUBQ) and mono-ubiquitylated (mUBQ) ELK-1 species. (**D**) HeLa cells were transfected with His.Tyg.UBQ and HA.ELK-1 as indicated before 24-h serum starvation and stimulation with TPA as indicated. WCEs were subjected to IMAC. Bound proteins were analysed by SDS-PAGE (7.5%) and immunoblotting. Lower panels: WCEs analysed for expression and ERK activation. (**E**) WCEs from HEK293T cells transfected with expression vectors for His.Tyg.UBQ, HA.ELK-1 and V^12^-Ras, as indicated, were subjected to IMAC. Isolated proteins were analysed by SDS-PAGE (5–20%) and immunoblotting with the antibodies indicated. Lower panels: WCEs analysed for protein expression and phosphorylation as indicated. (**F**) HeLa cells were transfected with His.Tyg.UBQ and HA.ELK-1 as indicated, serum-starved for 24 h, pre-treated with U0126 (10 μM) and/or SB202190 (5 μM) and stimulated with TPA as indicated for 1 h. WCEs were processed and analysed as in panel (D). (**G**) HeLa cells were transfected with vectors for His.Tyg.UBQ and HA.ELK-1, ELK-1(S383/9A) or ELK-1(ΔD/FxLA) as indicated before 24-h serum starvation and stimulation with TPA for times indicated. WCEs were processed and analysed as in panel (E). (**H**) HeLa cells were transfected with vectors for ELK-1 (no tag) and His.Tyg.UBQ or a lysine-less UBQ mutant (K0) as indicated before 24-h serum starvation and stimulation with TPA for times indicated. WCEs were processed and analysed as in panel (D). In panels D, F, G and H, numbers below upper immunoblots indicate relative band intensity as determined by densitometry.

ELK-1 and its neuronal-specific, truncated isoform sELK can be poly-ubiquitinated to mediate their degradation by the proteasome (Supplementary Figure S1a) (29). However, when immobilized metal affinity chromatography (IMAC) was used in conjunction with his-tagged ubiquitin to harvest ubiquitinated proteins from HEK293T cells, the ELK-1 species detected had single or low molecular weight (MW) ubiquitin conjugates (Figure 1A, lane 3). These ELK-mUBQ conjugates were unaffected by proteasome inhibition (Figure 1A, compare lanes 3 and 6). Populations of endogenous ELK-mUBQ could also be isolated and were enriched upon expression of an ubiquitin mutant (L73P) that is refractory to deubiquitinase (DUB) activity (Figure 1B) (36). We also observed the formation of both poly- and mono-ubiquitinated ELK-1 conjugates in cell-free ubiquitination assays using HeLa nuclear extracts (Nxts) supplemented with recombinant E1 and E2 enzymes (33) (Figure 1C; see also Supplementary Figure S1b and c).

ELK-mUBQ conjugates were readily detected in serum-starved HeLa cells but their levels decreased following mitogen stimulation (Figure 1D). Expression of a constitutively active RAS or RAF allele starkly reduced ELK-mUBQ levels (Figure 1E and Supplementary Figure S1d). The MEK inhibitor U0126 attenuated mitogen-stimulated loss of ELK-mUBQ (Figure 1F, compare lanes 7 and 8) whereas the p38^mapk^ inhibitor SB202190 did not (Figure 1F, compare lanes 7 and 9). We also observed that ELK-1 mutants lacking ERK docking motifs (ΔD/FxLA) or key phosphorylation sites (S383, S389) underwent enhanced mono-ubiquitination and that their mUBQ levels remained unchanged after mitogen stimulation (Figure 1G). It seemed plausible that ELK-mUBQ species might be intermediates in poly-ubiquitin chain formation. However, a lysineless ubiquitin (UBQ.K0) unable to build ubiquitin chains formed a unitary ELK-mUBQ conjugate (Supplementary Figure S1e), implying that ELK-mUBQ isoforms involve a single lysine modified with a ubiquitin mono-, di- or trimer. The ELK-1-UBQ.K0 conjugate was also lost upon mitogen stimulation (Figure 1H, lanes 7–10), suggesting that removal of mono-ubiquitin rather than chain extension and degradation is likely to account for ELK-mUBQ loss. Together these data indicate that ELK-1 can be mono-ubiquitinated in mitogen-starved cells and that the modification is removed upon ERK signalling and ELK-1 phosphorylation.

### ELK-1 is mono-ubiquitinated on K35 in the ETS domain

**Figure 2.**
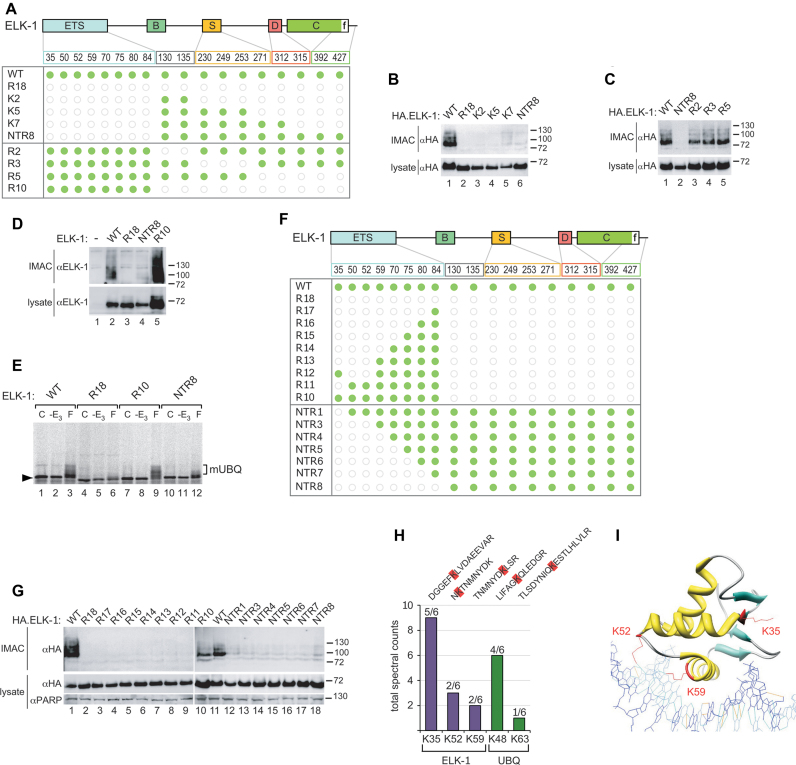
ELK-1 is mono-ubiquitinated in the ETS domain, predominantly on lysine 35. (**A**) ELK-1 domain structure (ETS domain; B = SRF interaction; S = SUMO consensus sites; D = MAPK docking; C = transactivation domain; f = ERK docking) indicating lysine distribution. The matrix represents lysines present in ELK-1 mutants as green discs and arginine substitutions as open grey circles. (**B–D**) WCEs from HEK293 cells transfected with expression vectors for His.Tyg.UBQ and HA.ELK-1, or the mutants indicated were subjected to IMAC. Isolated proteins were analysed by SDS-PAGE (7.5%) and immunoblotting with the antibodies indicated. Lower panels: WCEs analysed for ELK-1 expression. (**E**) ^35^S-labelled ELK-1 and mutants indicated were incubated alone (C), with E1, E2, UBQ and UA but without Nxt (-E_3_) or with all components (F) as indicated. Reactions were separated by SDS-PAGE (7.5%) and analysed by phosphor-imaging. Bracket indicates ELK-mUBQ species. (**F**) As in (A). (**G**) WCEs from HEK293 cells transfected with expression vectors for His.Tyg.UBQ and HA.ELK-1 or mutants indicated were processed and analysed as in (B–D). (**H**) ELK-1.His was isolated from HEK293T lysates by IMAC, subjected to tryptic digestion on beads and LC-MS/MS analysis (see ‘Materials and methods’ section). Bars represent total spectral counts for each peptide from six independent analyses. Peptide sequences are show above bars with di-gly remnant modified lysines (K+114) highlighted in red. (**I**) Structure of ELK-1 ETS domain indicating ubiquitinated lysines (red) in relation to bound DNA (adapted from (38).

To identify ubiquitination sites in ELK-1, we used mutants in which lysines had been substituted with arginines (Figure 2A). Elimination of lysines in the ETS domain abolished mono-ubiquitination (Figure 2B), whereas removal of lysines outside the ETS domain did not (Figure 2C). Removal of all 10 lysines outside the ETS domain (R10) caused significant ELK-1 accumulation (Figure 2D, lane 5). These data indicate that the mono-ubiquitination site(s) resides in the ETS domain of ELK-1 and imply that one or more C-terminal lysines might serve as sites of poly-ubiquitination. *In vitro* ubiquitination assays also indicated that ELK-1 was mono-ubiquitinated within the ETS domain (Figure 2E).

To map mono-ubiquitination site(s) within the ETS domain, we made multiple lysine substitutions in ELK-1 or re-introduced lysines into the R18 mutant (Figure 2F). Only WT and R10 versions of ELK-1 were mono-ubiquitinated and mutants R11 and NTR1 (Figure 2G, lanes 9 and 12) identified K35 as the probable site of mono-ubiquitination. We saw no evidence of R11 or NTR1 accumulation, unlike mutant R10 (Figure 2G, lane 10), a further indication that K35 is not a site of poly-ubiquitination. Mutant R12 was also not modified, implying that K50 and/or K52 are also required for ELK-1 mono-ubiquitination (see below).

To corroborate the functional mapping data, we used LC-MS/MS to identify modified lysines directly by di-glycine remnant mapping (Supplementary Figure S2) (37). We detected ELK-1 peptides with a di-glycine stub on K35 in multiple experiments. K52 and K59 di-glycine remnants were also detected but less frequently (Figure 2H). It is noteworthy that K52 and K59 both contact bound DNA (Figure 2I) (38). We occasionally detected K48 ubiquitin linkages, suggesting that ELK-mUBQ could include some short chain isoforms. Together, the functional mapping and MS/MS data identify K35 as the predominant mono-ubiquitination site in ELK-1.

### Ternary complex formation promotes ELK-1 mono-ubiquitination

**Figure 3.**
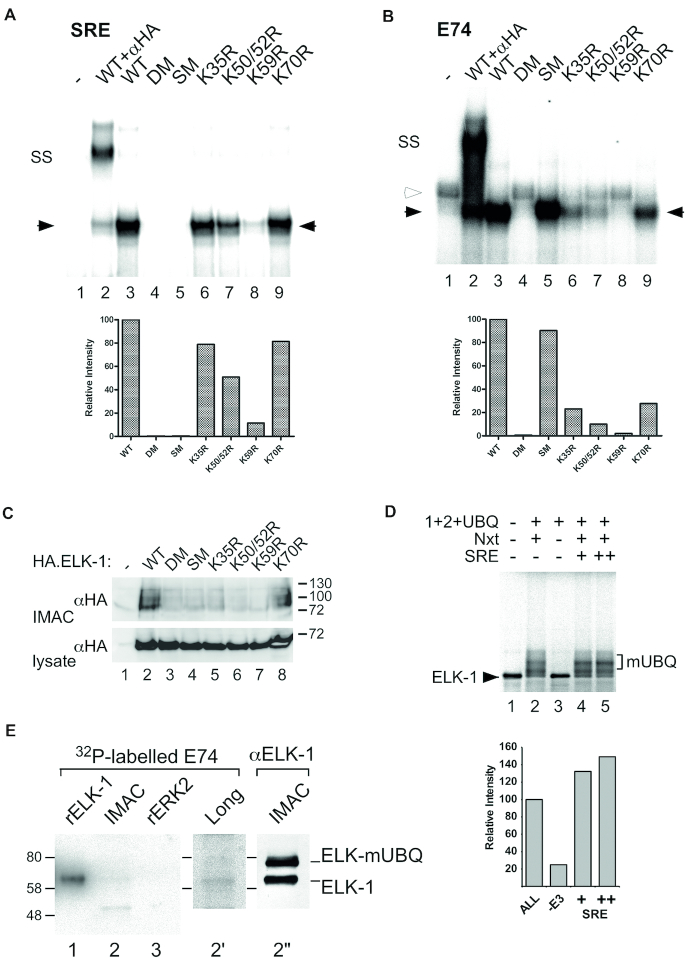
ELK-1 mono-ubiquitination requires ternary complex formation but blocks DNA binding. (**A**) HA.ELK-1 and mutant proteins indicated were incubated with a radio-labelled SRE probe and recombinant core^SRF^. Complexes were resolved by electrophoresis and visualized by phosphor-imaging. Solid arrowheads indicate ELK-1/E74 complexes; SS indicates super-shift obtained with anti-HA antibody (lane 2). Histogram (below) shows densitometric analysis of ELK-1/DNA complexes (solid arrows) in lanes 3–9 with ELK-1 set at 100%. (**B**) As in (A) except that complexes were formed with a radio-labelled E74 probe; open arrowhead indicates non-specific band seen in WCE from control cells (lane 1). (**C**) WCEs from HEK293 cells transfected with expression vectors for His.Tyg.UBQ and HA.ELK-1 or mutants indicated were subjected to IMAC. Isolated proteins were analysed by SDS-PAGE (7.5%) and immunoblotting with an anti-HA antibody. Lower panel: WCEs analysed for ELK-1 expression. (**D**) ^35^S-labelled ELK-1 was incubated alone (lane 1), with E1, E2, UBQ and UA (lanes 2–5) and with or without Nxt and an SRE oligonucleotide duplex as indicated. Reactions were separated by SDS-PAGE (7.5%) and analysed by phosphor imaging. Bracket indicates ELK-mUBQ species. Histogram (right) shows densitometry of mono-ubiquitinated species in lanes 2–5 with lane 2 set at 100%. (**E**) Lysate from HEK293T cells transfected with expression vectors for His.UBQ(L73P) and HA.ELK-1 was subjected to IMAC. Recombinant ELK-1, ERK2 (Supplementary Figure S5) and IMAC samples were separated by SDS-PAGE (5–20%) transferred to nitrocellulose, renatured and incubated with radio-labelled E74 probe. Lane 2′ shows longer exposure of lane 2. ELK-1 and ELK-mUBQ presence in IMAC sample was confirmed by immunoblotting with anti-HA antibody (lane 2′′).

As K52 and K59 in the ETS domain of ELK-1 directly contact bound DNA, we examined mono-ubiquitination in relation to DNA binding. We compared SRF-dependent binding to the *CFOS* SRE (39), direct binding to the high affinity E74 site (40) and levels of mono-ubiquitination of several ELK-1 lysine mutants and two previously characterized DNA binding-deficient mutants (29). ELK-1-SM (L158P/Y159A) is mutated in the B-domain and cannot bind SRF but has an intact ETS domain allowing E74 binding; ELK-1-DM (R65A/Y66F) is mutated in the ETS domain and cannot bind DNA. In electrophoretic mobility shift assays (EMSAs), the effects of mutations on SRF-assisted and direct DNA binding were similar (Figure 3A and B; Supplementary Figure S3) except for SM, which has an intact ETS domain allowing E74 binding. Thus, for different reasons, neither ELK-1-SM nor ELK-1-DM formed a ternary complex at the SRE (Figure 3A, lanes 4 and 5) and although both contain all 18 lysine residues, neither mutant was mono-ubiquitinated (Figure 3C, lanes 3 and 4). This suggests that ternary complex formation may facilitate mono-ubiquitination of ELK-1. Consistent with this idea, mono-ubiquitination of ELK-1 in our *in vitro* ubiquitination system increased upon inclusion of an oligonucleotide SRE duplex (Figure 3D). These data imply that weak ternary complex formation associated with mutants K50/52R and K59R (Figure 3A, lanes 7 and 8) may suppress their mono-ubiquitination (Figure 3C, lanes 6 and 7). In contrast, K35R and K70R both form ternary complexes with approximately 80% efficiency but only K70R is mono-ubiquitinated (Figure 3C, lane 8), consistent with the identification of K35 as the predominant mono-ubiquitination site in ELK-1.

### Mono-ubiquitination of ELK-1 impairs DNA binding

Where lysine substitutions in the ELK-1 ETS domain destabilize DNA binding, ubiquitin conjugates could have a similar effect. To test this notion, we used the his-tagged, DUB-resistant ubiquitin mutant (L73P) (36) to increase the level of ELK-mUBQ in HEK293T cells, which we sought to isolate by IMAC and test in EMSAs. However, post-IMAC ELK-mUBQ samples consistently contained unmodified ELK-1, which we put down to previously reported dimer formation (29,41). To circumvent this problem, we isolated similar amounts of ELK-1 and ELK-mUBQ from lysates under native conditions and performed a southwestern assay to compare their DNA binding in parallel. After SDS-PAGE and transfer to membrane, successful re-folding of immobilized protein was confirmed with recombinant ELK-1, which readily bound an E74 probe, whereas binding to renatured ERK was absent (Figure 3E, lanes 1 and 3). Significantly, although ELK-1 and ELK-mUBQ were present in almost equal amounts on the membrane (lane 2″), ELK-mUBQ bound approximately 4-fold less E74 probe than ELK-1 (lanes 2 and 2′). This result suggests that mono-ubiquitination of the ELK-1 ETS domain interferes with DNA binding.

### Absence of mono-ubiquitination potentiates ELK-1 transcriptional activity

**Figure 4.**
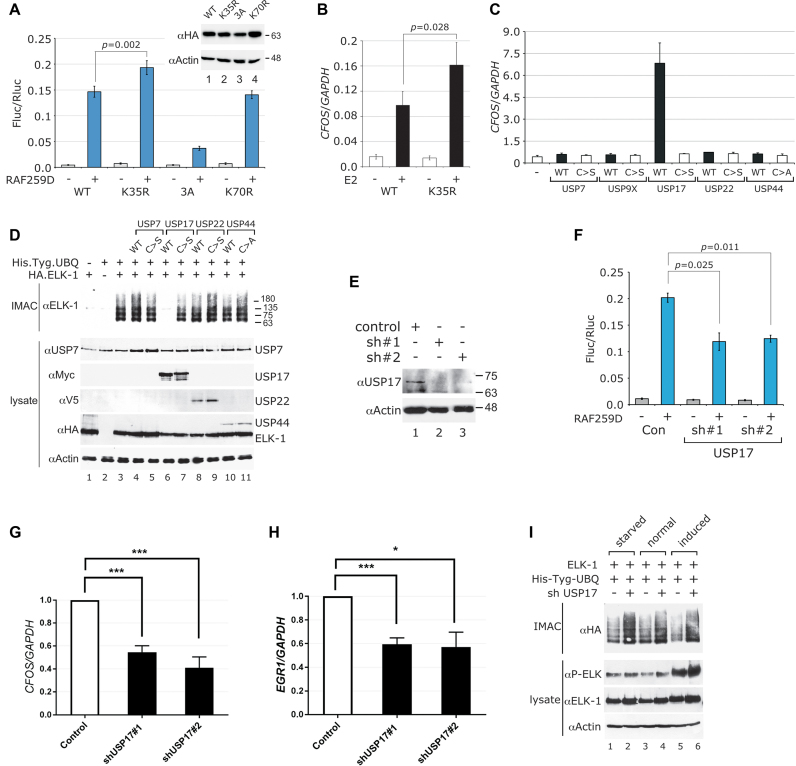
USP17 reverses mono-ubiquitination to augment ELK-1 transcriptional activity. (**A**) HEK293T cells were transfected with SRE-Fluc and control Rluc reporters, HA.ELK-1 or the mutants indicated and RAF259D (+) or vector control (-). After 48 h, cells were harvested and luciferase expression was analysed. Inset shows ELK-1 expression. Results are from three biological repeats and each performed in triplicate. (**B**) HEK293T cells were transfected with vectors for HA.ELK-1 or ELK-1(K35R) and BXB-ER, an oestrogen-inducible, active RAF-1. Cells were serum starved for 24 h before stimulation with estradiol (2 μM) or mock treatment. After 2 h, RNA was prepared for analysis by qRT-PCR. Results are from three biological repeats and each performed in triplicate. (**C**) HEK293T cells were transfected with vectors for WT or catalytically inactive DUBs, in which the catalytic cysteine is substituted with serine (C>S) or alanine (C>A), as indicated. RNA was isolated after 48 h and analysed by qRT-PCR. *CFOS* mRNA levels are normalized to *GAPDH* and represent means from three independent experiments, each assayed in triplicate. (**D**) WCEs from HEK293T cells transfected with expression vectors for His.Tyg.UBQ, HA.ELK-1 and active or inactive DUBs as indicated were subjected to IMAC. Isolated proteins were analysed by SDS-PAGE (5–20%) and immunoblotting. Lower panels: WCEs analysed for DUB and ELK-1 expression with antibodies indicated (left). (**E**) HEK293T cells were transfected with USP17 sh#1 and sh#2 vectors or vector control as indicated. USP17 levels were determined by SDS-PAGE (5–20%) and immunoblotting. (**F**) HEK293T cells were transfected with SRE-Fluc and control Rluc reporters, HA.ELK-1, the shRNA vectors indicated and RAF259D (+) or vector control (−). After 48 h, cells were harvested and luciferase expression was analysed. Results are from three biological repeats and each performed in triplicate. (**G** and **H**) HEK293T cells were transfected with shRNA vectors for USP17 or vector control as indicated. After 48 h, RNA was prepared and *CFOS* and *EGR1* mRNA were analysed by qRT-PCR. Levels are presented as fold ratios to *GAPDH* with controls normalized to 1 and represent means from four independent experiments, each assayed in triplicate. (**I**) WCEs from HEK293T cells transfected with expression vectors for His.Tyg.UBQ, HA.ELK-1 and control or USP17 shRNA vector as indicated were incubated either in full medium, serum-starved for 24 h or starved and stimulated with TPA for 40 min. WCEs were subjected to IMAC and isolated proteins were analysed by SDS-PAGE (5–20%) and immunoblotting with an anti-HA antibody. Lower panels: WCEs analysed for ELK-1 expression and phosphorylation. All graphs show significance as defined in Materials and Methods.

We used ELK-1 mutants to explore the potential impact of mono-ubiquitination on ELK-1 transcriptional activity. In luciferase reporter assays, ELK-1 was activated efficiently (approximately 30-fold) with an active RAF-1 that mimics mitogen signalling and this activation was blunted by alanine substitutions at key ERK phosphorylation sites [ELK-1(3A)]. The transcriptional activity of ELK-1(K35R) (i.e. mutant NTR1) was significantly elevated over ELK-1 (Figure 4A), whereas ELK-1(K70R), with SRE binding comparable to ELK-1(K35R) but normal levels of mono-ubiquitination (see Figure 3A–D), showed no change in activity. Mutants ELK-1(K50/52R) and ELK-1(K59R) were not evaluated because of their severely impaired ternary complex formation (see Figure 3A). ELK-1 mono-ubiquitination also affected endogenous gene expression. In cells transfected with an oestrogen-inducible active RAF-1 (RAF-ER) (42), estradiol stimulated significantly higher expression of *CFOS*, an established ELK-1 target gene, when ELK-1(K35R) was expressed instead of ELK-1 (Figure 4B). These findings suggest that mono-ubiquitination of K35 decreases the transcriptional activity of ELK-1, most likely by destabilizing DNA binding.

### USP17 (DUB-3) de-ubiquitinates ELK-1 to up-regulate target gene expression

Ubiquitin conjugation is reversed by deubiquitinases (DUBs) (43), so ectopic expression of a DUB with specificity for ELK-1 might increase ELK-1-dependent gene expression. Consideration of GO terms for all human DUBs identified several candidates with profiles similar to ELK-1, all of which belonged to the USP family of DUBs. Among those tested, only USP17 significantly increased ELK-1 reporter activity, whereas its catalytically inactive counterpart (C>S) had no effect (Supplementary Figure S4a). We then assessed the effect of ectopic DUB expression on transcription of the ELK-1 target gene *CFOS*. USP17 expression markedly increased endogenous *CFOS* mRNA levels whereas catalytically inactive USP17 and other DUBs did not (Figure 4C and Supplementary Figure S4b). In agreement with these findings, USP17 expression caused the loss of ELK-mUBQ from cells whereas levels were unaffected by a catalytically inactive USP17 or other DUBs (Figure 4D). In complementary experiments, we used two previously characterized shRNA vectors to deplete USP17 from HEK293T cells (44,45) (Figure 4E). As anticipated, USP17 depletion decreased ELK-1 reporter activity (Figure 4F), and significant reductions in *CFOS* and *EGR1* mRNA were seen compared to control cells (Figure 4G and H). Furthermore, USP17 depletion increased ELK-mUBQ levels in serum-starved, normally growing and mitogen-stimulated cells (Figure 4I). These data show that USP17 can de-ubiquitinate ELK-mUBQ and increase the expression of ELK-1 target genes.

### USP17 interacts directly with ELK-1 and countermands ELK-1 nuclear export

**Figure 5.**
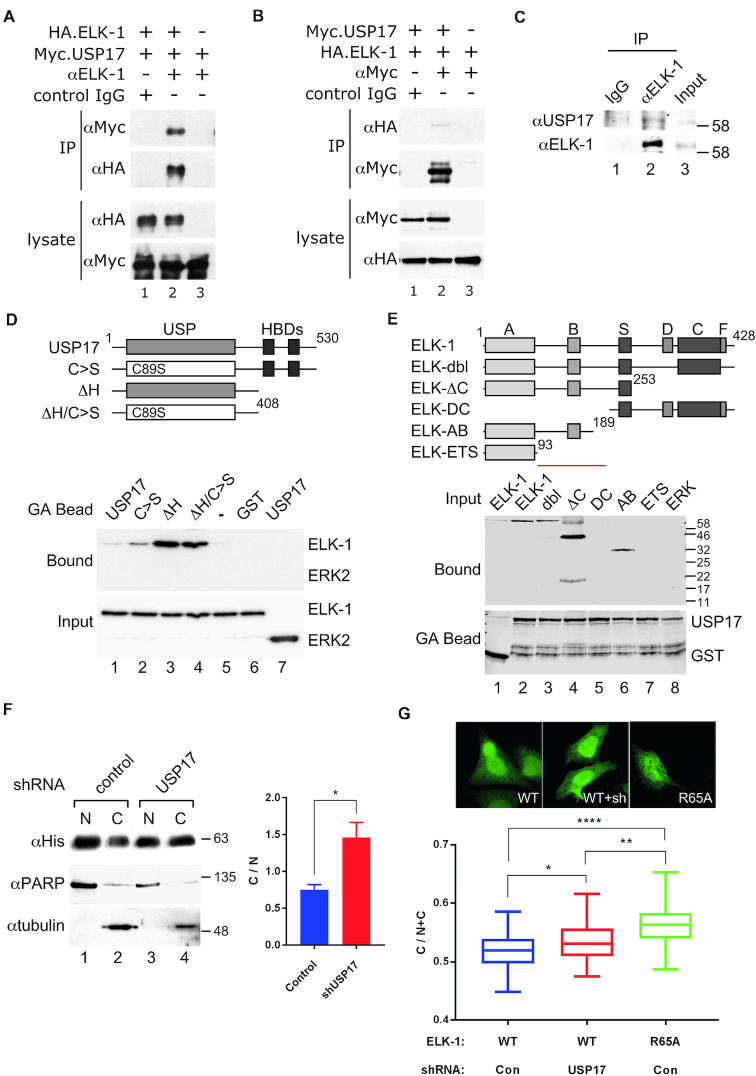
USP17 acts directly on ELK-1 to reverse mono-ubiquitination and nuclear export. (**A**) WCEs were prepared from HEK293T cells transfected with vectors for HA.ELK-1 and/or catalytically inactive Myc.USP17. Immunoprecipitates were collected with control IgG, or αElk-1 antibody and analysed by SDS-PAGE (5–20%) and immunoblotting as indicated. (**B**) As in (A) except that immunoprecipitates were collected with control IgG, or αMyc (USP17) antibody. (**C**) WCEs were prepared from DU145 cells and immunoprecipitates were collected with control IgG or αELK-1 antibody and analysed by SDS-PAGE (5–20%) and immunoblotting as indicated. (**D**) GST or GST-USP17 fusions (see diagram) on glutathione agarose beads were incubated with ELK-1 or ERK2. Input and bound proteins were analysed by SDS-PAGE (8%) and immunoblotting with an anti-His antibody. (**E**) GST or GST-USP17(ΔH) on glutathione agarose beads were incubated with ELK-1 deletion mutants (see diagram, key as in Figure 2A legend) or ERK2. Bound proteins and fusion proteins on beads were analysed by SDS-PAGE (8%) and immunoblotting with an anti-His antibody. Red bar denotes region in ELK-1 required for USP17 interaction. (**F**) Nuclear (N) and cytosolic (C) fractions were prepared from HEK293T cells transfected with vectors for ELK-1.His and control or USP17 shRNA, submitted to IMAC and analysed by SDS-PAGE (5–20%) and immunoblotting with antibodies indicated. Graph shows C/N distribution ratios. Results are from three biological repeats. (**G**) HeLa cells transfected with vectors for ELK-1-GFP, ELK-1(R65A)-GFP and control or USP17 shRNA, as indicated, were fixed and analysed by high content confocal imaging. Data shown (*n* = 48) are from one of three experiments with similar, statistically significant results (Student’s *t*-test); significance as defined in Materials and Methods. Inset panels show indicative GFP distribution.

ELK-1 and USP17, when expressed ectopically, readily co-immunoprecipitated from HEK293T cell lysates (Figure 5A and B). Endogenous ELK-1 and USP17 also co-immunoprecipitated from DU145 prostate cancer cells, in which USP17 expression is elevated (Figure 5C). Their interaction appears to be direct because recombinant GST-USP17 bound to ELK-1 *in vitro* and deletion of the C-terminal hyaluronan binding domains (46) from USP17 enhanced this interaction (Figure 5D and Supplementary Figure S5a). Analysis of binding to a series of ELK-1 deletion mutants indicated that USP17 recognizes a motif located between the ETS (A) and S domains, as ELK-1 deletions lacking this region did not bind to USP17 (Figure 5E and Supplementary Figure S5b).

Ubiquitination contributes to Mdm2-dependent nuclear export of p53 (47,48), so we looked into whether mono-ubiquitination levels affected the cellular distribution of ELK-1. We found that USP17 knockdown, which increased ELK-mUBQ levels (Figure 4I), caused a net redistribution of ELK-1 from nucleus to cytoplasm (Figure 5F). Cell fractionation also revealed that ELK-mUBQ was mostly cytosolic (Supplementary Figure S5c). Furthermore, USP17 knockdown in HeLa cells increased the cytoplasmic distribution of an ELK-1-GFP fusion, in line with an ELK-1(R65A)-GFP fusion that no longer binds to DNA (Figure 5G). Enhanced nuclear export of DNA-binding-defective ELK-1 mutants had been observed previously (29). Collectively, these data indicate that mono-ubiquitination suppresses ELK-1 transcriptional activity through impaired DNA binding and consequent cytoplasmic redistribution of ELK-mUBQ. Conversely, USP17 interacts directly with ELK-1 to remove mUBQ conjugates, suppress nuclear export and augment ELK-1 transcriptional activity.

### USP17 promotes ELK-1 target gene expression and cell proliferation

**Figure 6.**
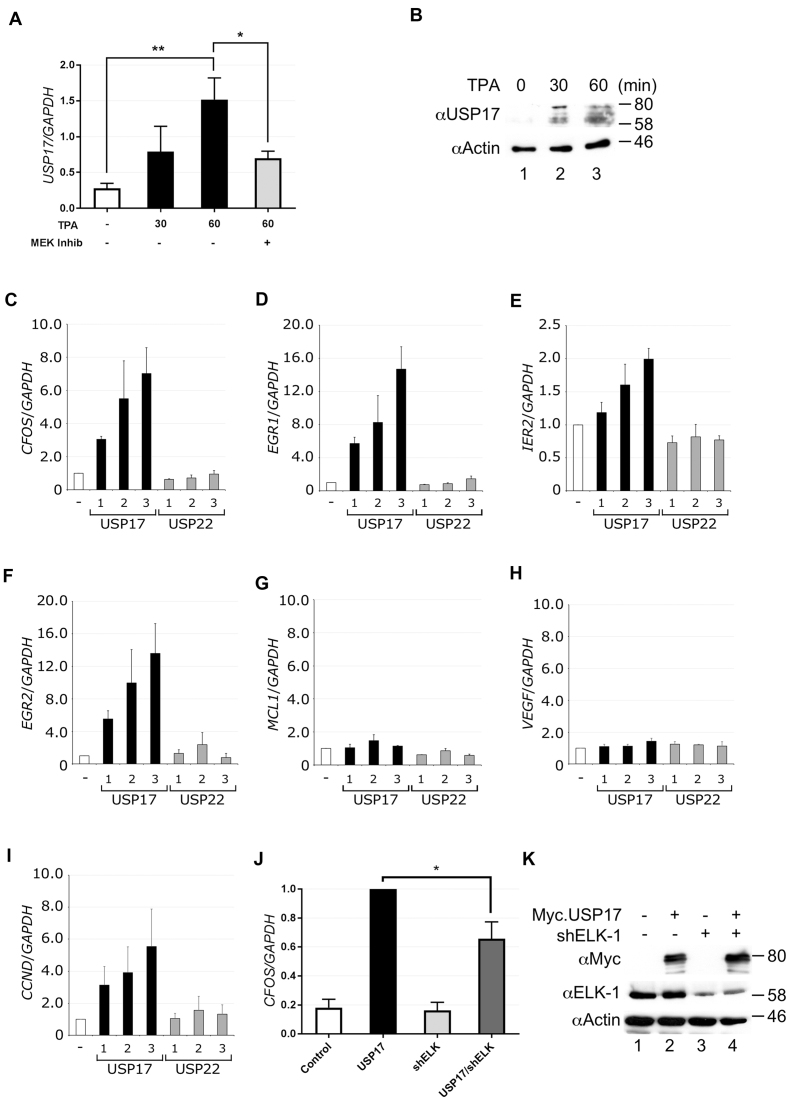
USP17 expression is mitogen inducible and up-regulates ELK-1 target genes. (**A**) HeLa cells were treated with TPA and MEK inhibitor U0126 as indicated. RNA was isolated and analysed by qRT-PCR for *USP17* mRNA. Results are from four biological repeats and each performed in triplicate. (**B**) Lysates from HeLa cells treated with TPA for 0, 30 and 60 min were analysed by SDS-PAGE (5–20%) and immunoblotting with antibodies indicated. (**C–I**) HEK293T cells were transfected with increasing amounts of vector for USP17 or USP22. RNA was isolated after 48 h and analysed by qRT-PCR for *CFOS* (C), *EGR1* (D), *IER2* (E), *EGR2* (F), *MCL1* (G), *VEGF* (H) or *CCND1* (I) mRNA. All mRNA levels were normalized to *GAPDH* and represent means from three independent experiments, each assayed in triplicate. (**J**) HEK293T cells were transfected with Myc.USP17 and shRNA for ELK-1 as indicated. RNA was isolated after 48 h, and *CFOS* mRNA was analysed by qRT-PCR. Levels are presented as fold ratio of *CFOS/GAPDH* with USP17 normalized to 1 and represent means from three independent experiments, each assayed in triplicate. (**K**) Lysates from HEK293T cells from (J) were analysed by SDS-PAGE (5–20%) and immunoblotting with antibodies indicated. Significance is defined in Materials and Methods.

The human *USP17* gene is cell cycle regulated and its expression in G1 promotes G1-S progression (31). We found that *USP17* expression was stimulated by TPA and suppressed by MEK inhibition (Figure 6A and B). Although delayed with respect to expression of *CFOS* (Supplementary Figure S6), *USP17* expression correlated with the observed time frame of ELK-1 de-ubiquitination (see Figure 1D). Ectopic expression of USP17 substantially increased mRNA levels of multiple ELK-1 target genes (*CFOS, EGR1, EGR2, IER2*) but not control genes (*MCL1, VEGFA*), whereas expression of USP22 had no such effect (Figure 6C–H). Increased levels of *cyclin D1* (*CNND1*) mRNA were also observed in cells transfected with USP17 but not with USP22 (Figure 6I). To confirm that this increase in gene expression involved ELK-1, we used short hairpin RNA (shRNA) to deplete endogenous ELK-1 and found that this significantly reduced USP17-mediated *CFOS* expression (Figure 6J and K). These data are consistent with the notion that USP17 promotes mitogen-induced, ELK-1-dependent gene expression.

**Figure 7.**
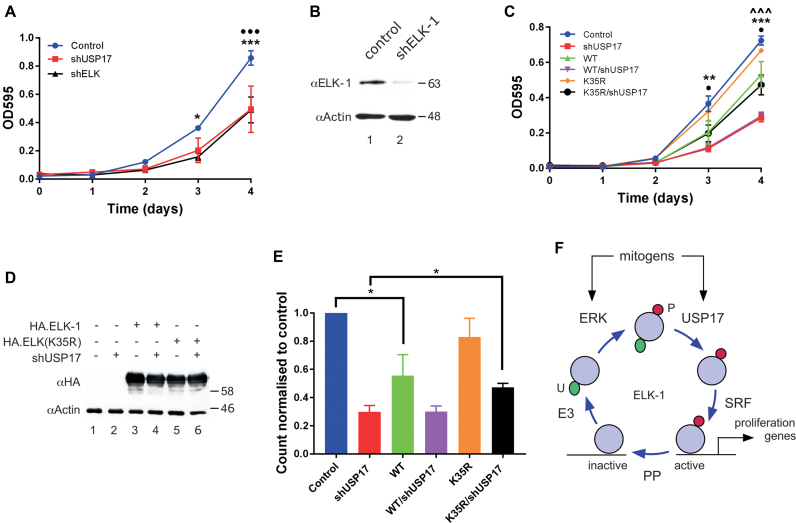
USP17-mediated de-ubiquitination of ELK-mUBQ promotes cell proliferation. (**A**) HEK293T cells were transfected with shUSP17#1, shELK-1 or vector control as indicated. One day post transfection cells were seeded into 96-well plates and proliferation was assessed every 24 h by MTT assay. Data are averages of three independent experiments in which each point is the average from four measurements; error bars show SEM. • = control v. USP17; * = control v. ELK-1. (**B**) HEK293T cells were transfected with vector control or ELK-1 shRNA vector as indicated. ELK-1 levels were determined by SDS-PAGE (5–20%) and immunoblotting. (**C**) As in (A) except that cells were transfected with vectors for shUSP17#1, ELK-1(WT), ELK-1(K35R) or vector controls as indicated. Data are averages of three independent experiments; error bars show SEM: • = WT v. K35R; * = control v. WT; ∧ = shUSP17 v. K35R/shUSP17. (**D**) HEK293T lysates from (c) were analysed by SDS-PAGE (5–20%) and immunoblotting with antibodies indicated. (**E**) As in (C) except that cells were seeded into 24-well plates and counted on day 4. Data are averages of three independent experiments; error bars show SEM, with control normalized to 1. Significance is defined in Materials and Methods. (**F**) Model for ELK-1 regulation involving phosphorylation (P) and removal of mono-ubiquitin (U). SRE-binding promotes ELK-1 mono-ubiquitination and nuclear export. Mitogens stimulate ERK phosphorylation of ELK-1 and *de novo* expression of USP17. USP17 reverses mono-ubiquitination to increase availability of active ELK-1 during G1. In concert with SRF, active ELK-1 stimulates expression of target genes to drive G1 progression and cell proliferation. Protein phosphatases (PP) can terminate ELK-1 activity.

Depletion of USP17 has been shown to inhibit HeLa cell proliferation (31). We confirmed this observation in MTT assays (Supplementary Figure S7a). Similarly, we found that USP17 knockdown inhibited HEK293T cell proliferation (Figure 7A). Furthermore, depletion of ELK-1 had the same negative effect on HEK293T cell proliferation as USP17 knockdown, confirming a positive regulatory role for ELK-1 during proliferation in a ubiquitous human cell model (Figure 7A and B; Supplementary Figure S7b).

We then tested whether the slower proliferation associated with USP17 loss could be rescued by ELK-1. Ectopic expression of ELK-1 caused a significant reduction in HEK293T proliferation, whereas ELK-1(K35R) appeared to have little effect (Figure 7C and D). However, ELK-1(K35R) partially rescued the decrease in proliferation associated with loss of USP17, whereas ELK-1 had no remedial effect. A significant rescue of USP17 knockdown by ELK-1(K35R), but not ELK-1, was also seen independently in cell counting assays (Figure 7E). Collectively, these data indicate that mono-ubiquitination places a constraint on ELK-1 activity that is removed in proliferating cells by USP17. We conclude that ELK-1 is a *bona fide* client of USP17 and that the positive impact of USP17 on cell proliferation is mediated in part through de-ubiquitination of ELK-1.

## DISCUSSION

In response to mitogens, ELK-1 up-regulates a set of target genes to promote and support cell proliferation (1–3). While the underlying role of ELK-1 phosphorylation has been explored in depth, how other post-translational modifications affect ELK-1 function is less well understood. Here, we have shown that mono-ubiquitination of K35 in ELK-1 suppresses its activity and that mitogens stimulate USP17 to reverse this modification, thereby augmenting ELK-1 transcriptional responses to ERK signalling. Our findings indicate that USP17 acts on the ERK–ELK-1 axis to promote cell proliferation and provide new mechanistic insight into the contribution of USP17 to aggressive phenotypes associated with diverse cancers (30–32).

### Impact of mono-ubiquitination on ELK-1 function

Several lines of evidence confirmed the conjugation of mono-ubiquitin to the amino-terminal ETS domain of ELK-1. Mapping of modification sites using lysine substitution mutants was ambiguous, due possibly to promiscuity of the ubiquitin ligase(s) responsible. Moreover, scrutiny of lysines involved in DNA binding (38) was complicated because ELK-1 mutants defective for SRF-mediated DNA-binding were refractory to mono-ubiquitination (Figure 3A and C). Interestingly, ELK-1-DM and ELK-1-SM mutants with a full complement of lysines were also not modified, implying that ternary complex formation with SRF promotes ELK-1 mono-ubiquitination. SRF present in HeLa nuclear extracts may have contributed to the enhanced *in vitro* mono-ubiquitination of ELK-1 seen with SRE duplex DNA. Ubiquitination of proteins associated with DNA is known and exemplified by RNF8 acting in the DNA damage response (49,50). Alternatively, shorter nuclear residence times associated with DNA binding-defective ELK-1 mutants may reduce their availability to a nuclear ubiquitin E3 ligase (29).

LC-MS/MS-based di-glycine remnant mapping (37) unequivocally identified K35 as the major mono-ubiquitination site on ELK-1. K35 lies in β2 remote from helix α3 (Figure 2I) and a K35R substitution is compatible with ternary complex formation (Figure 3A and C). Minor ubiquitination sites K52 and K59 lie within the α2/α3 loop and α3 helix respectively and contact bound DNA (38). Interestingly, ternary complex formation, which our data suggest promotes mono-ubiquitination of ELK-1, appears to be incompatible with modification at these two sites. Conversely, mono-ubiquitination of ELK-1 appears to impede DNA binding. K52R and K59R substitutions disrupt DNA contacts (Figure 3A and B) and ubiquitination of either lysine would introduce steric interference. How mono-ubiquitination of K35 might affect DNA binding is less readily apparent. Studies on Ets-1 revealed that contacts between α1 of the ETS domain and two vicinal Ets-1 helices allosterically inhibit DNA binding (51). Conceivably, ubiquitin conjugation to K35 on this surface of the ELK-1 ETS domain could result in a similar allosteric effect, destabilizing DNA binding and facilitating the ubiquitination of K52 and K59. Unequivocal evidence for these effects will require studies on homogeneous samples of ELK-mUBQ isoforms.

The increase in ELK-mUBQ and partial redistribution of ELK-1 to the cytoplasm upon USP17 depletion is consistent with impaired DNA binding, as noted earlier for DNA-binding-defective ELK-1 mutants (29). However, it could also reflect preferential engagement of the nuclear export machinery with ELK-mUBQ. Alternatively, modification of K52, which lies within a consensus nuclear localization signal (52), may impair ELK-1 nuclear import. On balance, mono-ubiquitination seems to diminish the transcriptional activity of ELK-1 by impairing DNA binding and promoting its cytoplasmic re-localization.

### Contribution of de-ubiquitination to ELK-1-dependent gene regulation

Approximately 95 mammalian DUBs serve to reverse the actions of ubiquitin E3 ligases (43). Several DUBs are known to include transcription regulators among their client proteins: USP7 (HAUSP) de-ubiquitylates Gli proteins to up-regulate gene expression downstream of Hedgehog signalling (53); USP9X de-ubiquitylates SMAD4 to modulate TGFβ signalling (54). Relationships between transcription factors and DUBs can be complex: for example USP7, USP12, USP14, USP22 all appear to regulate the stability and function of androgen receptor (AR) in prostate cancer (55–58). As our search for an ELK-1 DUB focused on enzymes whose biological characterization, tissue distribution and intracellular location overlapped with those of ELK-1, we cannot rule out the possibility that other DUBs besides USP17 regulate ELK-1 activity.

ELK-1 and USP17 formed complexes in cells, they interacted directly *in vitro* and USP17 expression caused ELK-1 de-ubiquitination, correlating with increased transcriptional activity, the specific expression of multiple ELK-1 target genes and the downstream cell cycle regulator *CCND1* (cyclin D1) (59). Conversely, the higher ELK-mUBQ levels observed upon USP17 knockdown correlated with reduced ELK-1 activity and target gene expression. Depletion of ELK-1 substantially retarded HEK293T cell proliferation, as did depletion of USP17, in line with previous reports (31), and expression of ELK-1(K35R), which we showed to be hypo-modified and hyper-active, substantially reversed the effect of USP17 depletion, linking modification of K35 in ELK-1 to observed cellular phenotypes. Although an ELK-1(K35N) mutation has been observed in an ovarian carcinoma (60), this is the first report to identify mono-ubiquitination of K35 as a modulator of ELK-1 activity and USP17 as the enzyme responsible for its removal.

### Role of USP17 in cell proliferation and cancer

The *USP17* gene (*DUB3*) has a highly variable copy number within the human genome (61). *USP17* expression is cytokine responsive, cell cycle regulated and linked to G1/S progression (30–32). Other USP17 substrates have been identified. Elevated USP17 expression has been reported to promote de-ubiquitination of RCE1, interfere with membrane localization of H-RAS and suppress MEK/ERK signalling (62). USP17 expression also caused de-ubiquitination and stabilization of CDC25A, resulting in replication stress and activation of the DNA damage response (30). Furthermore, USP17 mediated de-ubiquitination of H2AX and DEC1 has been shown to delay DNA damage checkpoint recovery (44,63). These phenomena correlate USP17 overexpression with stalled cell proliferation.

USP17 is nonetheless strongly implicated in promoting cell proliferation. In tumour-derived cells and biopsies, USP17 expression was found to be elevated (30,31). In ovarian cancer and NSCLC, levels of *USP17* expression correlated positively with tumour progression (32,64). This role may be synergistic with CDC25A, because elevated CDC25A levels in human cancer correlated positively with USP17 activity, and USP17 was shown to cooperate with H-RAS to transform NIH3T3 cells (30). USP17 also acts to stabilize SNAIL1 in metastatic breast cancers in a manner dependent on prior phosphorylation of USP17 by G1 cyclins CDK4/6 (65). Thus, USP17 bears hallmarks of a transforming oncogene and is able to promote tumour progression.

Activation of ELK-1 is contingent upon ERK phosphorylation at multiple sites in the C-terminal trans-activation domain (5–7). Phosphorylation of ELK-1 may promote USP17-mediated de-ubiquitination, because ELK-1 mutants refractory to ERK phosphorylation resisted de-ubiquitination in mitogen-stimulated cells. In the absence of evidence for a phospho-recognition motif in USP17 clients, it is tempting to infer that phosphorylation-induced conformational change promotes ELK-1 de-ubiquitination (66). As we confirm, mitogens also promote USP17 expression in G1 (31). In murine ES cells (mESCs) Esrrb, a component of the self-renewal machinery, up-regulates the expression of *Usp17* (67). Notably, down-regulation of *Usp17* in mESCs induced their spontaneous differentiation (68). Likewise, human ESCs from which ELK-1 was depleted ceased to proliferate and underwent differentiation (2). USP17-mediated de-ubiquitination of ELK-1 may therefore help to sustain ESC self-renewal as well as promoting G1 progression.

Individual knockdowns of USP17 and ELK-1 both impacted negatively on HEK293T cell proliferation. These effects were linked because expression of ELK-1(K35R) substantially rescued the loss of proliferation associated with USP17 depletion. The effectiveness of ELK-1(K35R) in these experiments is perhaps surprising given that ELK-1 is just one of several USP17 clients and, arguably, may be functionally redundant to ELK-3 and ELK-4 (8–10). The impact of USP17 acting on ELK-1 to augment ERK signalling is thus likely to vary in different cellular contexts. Nonetheless, our findings uncover an important functional relationship between USP17 and ELK-1 in its conserved role as ERK responder and activator of mitogenic gene expression (Figure 7F). Indeed, the impact of USP17 on ELK-1 function may extend beyond its relationship with ERK, given the recent discovery of a physical and functional cooperation between ELK-1 and androgen receptor in advanced prostate cancer (69,70). A next priority will be to identify the ubiquitin E3 ligase(s) specifically responsible for mono-ubiquitination of ELK-1.

## Supplementary Material

Supplementary Data

## Notes

Present address: Leo Kam Yuen Chow, TVM Capital China Ltd., Cyberport, Hong Kong SAR.

Present address: Jürgen Handwerger, G. Pohl-Boskamp GmbH, 25551 Hohenlockstedt, Germany.
