# Supplementary material for: De-ubiquitination of ELK-1 by USP17 potentiates mitogenic gene expression and cell proliferation
Source: Nucleic Acids Res. 2019 Mar 11;47(9):4495–508. doi: 10.1093/nar/gkz166 (PMC6511843; doi:10.1093/nar/gkz166)
Supplement: Supplementary Data [file gkz166_supplemental_files.zip › ELK MUBI SUPP INFO REV.pdf]

Charles Ducker el al

SUPPLEMENTARY INFORMATION

Table S1. Antibodies

ANTIBODY	CODE	SOURCE
ELK-1	H-160 (SC-22804)	Santa Cruz
ELK-1	I-20 (SC-355)	Santa Cruz
ElkC	rabbit polyclonal	Shaw Lab
ELK-1	E277	Abcam
phospho-ELK-1 (pS383)	B4 (SC-8406)	Santa Cruz
ERK	C14 (SC-154)	Santa Cruz
phospho-ERK	E4 (SC-7383)	Santa Cruz
HA	3F10 (11867423001)	Roche
His	MCA1396GA	ADB Serotec
V5	ABJ792	Millipore
Myc	SC-40	Sant Cruz
FLAG	MCA4764	ABD Serotec
Strep	E12-016	Enogene
USP7	A300-033AT	Bethyl
USP17/DUB-3	PA5-44961	Invitrogen
Actin	A2066	Sigma
PARP	550429	BD Biosciences
Tubulin	SC-8035	Santa Cruz

Table S2. Protein Expression and shRNA Plasmids

PROTEIN	VECTOR	SOURCE
His.ELK-1	pCMV5	Gille et al (1995)
HA.ELK-1	pcDNA3	Bob Hipskind, Montpellier
HA.ELK-1(3A)	pcDNA3	Bob Hipskind, Montpellier
pQE-ELK.1, ΔD, DC	pQE60	Gille et al (1995)
pQE-ELK ETS, AB	pQE60	Saxton et al (2016)
HA.Ubiquitin	pCMV5	Simon Dawson, Nottingham
Ubiquitin (K0)	pCMV5	Gu Wei, New York
Ubiquitin(L73P)	pcDNA3.1	This work
V^{12}-RAS	pCMV5	Melanie Cobb, Dallas TX
RAF-259D	pCMV5	Walter Kölch, Dublin
BXB-ER	pCMV5	Helge Steen, Shaw Lab
Firefly luciferase	pGL3 $(SRE)_3$	Bob Hipskind, Montpellier
Renilla luciferase	pGL4 hRluc UbC	Galbraith et al (2013)
USP7 (WT + C223S)	pcI	Roger Everett, Glasgow
USP9x (WT+C1566S)	pCMV TnT	Ralf Kittler, Dallas TX
USP17 (WT + C89S)	pcDNA3	Daniele Guardavaccaro, Utrecht
USP22 (WT + C158S)	pcDNA3.1 V5/Flag	Sharon Dent, Smithville TX
USP44 (WT + C282A)	pcDNA4 strep/HA	Neils Mailand, Copenhagen
psh USP17#1	pSUPER	Daniele Guardavaccaro, Utrecht
psh USP17#2	pSUPER	Daniele Guardavaccaro, Utrecht
psh ELK-1	pSUPER	This work
GST-USP17, C89S, ΔH	pGEX-KG	This work

Table S3. shRNA target sequences

psh USP17#1	NM_201402.2	GCAGGAAGATGCCCATGAA
psh USP17#2	NM_201402.2	GAATGTGCAATATCCTGAG
psh ELK-1	NM_001114132.2	GGCCTTGCGGTACTACTAT

Table S4. RT-PCR Probes and Primers

Gene	NCBI ID	Forward primer (5'-3')	Reverse primer (5'-3')	Taqman Probe (5'-3')
CFOS	NM_00525.2	ACTACCACTCACCCGCAGAC	GTGGGAATGAAGTTGGCACT	CCTGTCAACGCGCAGGACTTCTG
EGR1	NM_001964.2	CAGCACCTTCAACCCTCAG	CAGCACCTTCTCGTTGTTCA	CTACGAGCACCTGACCGCAGAGTCTT
EGR2	NM_000399.4	TTGACCAGATGAACGGAGTG	GCCCATGTAAGTGAAGGTCTG	AGGTGCAGAGACGGGAGCAAAG
IER2	NM_004907	AGTTCGGAATTTCGGTTCAA	GCCTGGAAGGGAAACTGA	CCTCGGATTGTTCCTGCGCA
MCL1	NM_021960.4	ACGGGTCACTACCCTCGAC	CCCATTGGCTTTGTGTCCT	TACCGGCAGTCGCTGGAGATTATCTC
VEGFA	NM_001025366	GCCCACTGAGGAGTCCAACAT	TGGCCTTGGTGAGGTTTGA	CCATGCAGATTATGCG
GAPDH	NM_002046.3	CTGCACCACCAACTGCTTAG	ACAGTCTTCTGGGTGGCAGT	CCCTGGCCAAGGTCATCCATG
USP17	NM_201402.2	AGAGCCCTCTTGCTGTGTTT	CCCTGCTAAACCTCTCTTCG	TGGCACCCTCGCTTCTCTGC

LEGENDS TO SUPPLEMENTARY FIGURES

Supplementary Figure S1

Confirmation of ELK-1 mono-ubiquitination *in vitro*

a) WCEs from HEK293 cells transfected with expression vectors for HA.UBQ and ELK-1.His were subjected to IMAC. Isolated proteins were analysed by SDS-PAGE (7.5%) and immunoblotting with the antibodies indicated. Where indicated cells were treated with MG132 (10μM) for 8h prior to harvest. Lower panels: WCEs analysed for ELK-1 and phospho-ELK-1 with the antibodies indicated.

b) ^{35}S-labelled ELK-1 or the lysine-less mutant (R18) were incubated with recombinant E1, recombinant E2, UBQ and ubiquitin aldehyde (UA) in the presence (F) or absence (-E3) of HeLa nuclear extract (Nxt) as indicated. Reactions were separated by SDS-PAGE (8%) and transferred to PVDF membrane, which was analysed by phosphor-imaging (lower panel) and subsequently probed with an anti-ELK-1 antibody (upper panel). Brackets indicate ELK-mUBQ species.

c) ^{35}S-labelled ELK-1 or the lysine-less mutant (R18) was incubated with recombinant E1, recombinant E2, Nxt, ubiquitin aldehyde (UA) and either UBQ or his-tagged UBQ as indicated. After incubation for 1h the reactions were divided into two fractions, one of which was subjected to IMAC to select ubiquitin conjugates. Pre- (lanes 2, 5, 8) and post-IMAC fractions (lanes 3, 6, 9) were separated by SDS-PAGE (8%) and analysed by phosphor-imaging. Radio-labelled ELK-mUBQ species are present in lane 3 but not in lanes 6 or 9.

Mitogen signalling induces loss of mono-ubiquitin

d) WCEs from HEK293T cells transfected with expression vectors for His-Tyg-UBQ, HA.ELK-1 and active RAF259D, as indicated, were subjected to IMAC. Isolated proteins were analysed by SDS-PAGE (5-20%) and immunoblotting with the antibodies indicated. Lower panels: WCEs analysed for protein expression and phosphorylation as indicated.

Lysine-less ubiquitin yields a single ubiquitin conjugate.

e) WCEs from HEK293T cells transfected with expression vectors for HA.ELK-1, His.Tyg.UBQ or His.Tyg.UBQ-K0 were subjected to IMAC. Isolated proteins were analysed by SDS-PAGE (5-20%) and immunoblotting with the antibodies indicated. Lower panels: WCEs analysed for protein expression as indicated.

Supplementary Figure S2

Di-glycine remnant mapping of ELK-1 mono-ubiquitination

a) Ubiquitin conjugation covalently links the C-terminal RGG motif of ubiquitin to acceptor lysines. Trypsinolysis leaves a residual di-glycine moiety on the modified lysine and is associated with C-terminal missed cleavage. MS/MS thus identifies daughter ions with m/z charge differences of 261 (K+114), as shown for an ELK-1 peptide encompassing amino acids 30-44.

b) ELK-1 ETS domain sequence showing secondary structure motifs and branched peptides detected (yellow) with modified lysines (red). Arrowheads indicate tryptic cleavage sites. No single peptide doubly modified at K52 and K59 was detected.

Supplementary Figure S3

ELK-1 mutant expression levels

Nuclear extracts used in Figure 5a and b prepared from HEK293 cells transfected with expression vectors for ELK-1 or mutants indicated examined by SDS-PAGE (7.5%) and immunoblotting with an ELK-1 antibody.

Supplementary Figure S4

Effect of DUB expression on ELK-1-dependent reporter expression.

a) HEK293T cells were transfected with SRE-Fluc and control Rluc reporters, expression vectors for ELK-1 and WT or catalytically inactive DUB, as indicated, along with RAF259D (+) or vector control (-). After 48h, cells were harvested and luciferase expression was analysed. Results are from three biological repeats each performed in triplicate.

b) Expression of WT and catalytically inactive (C>S) versions of USP7, USP17, USP22 and USP44 (C>A) in HEK293T cells. Equal aliquots of lysates from triplicate points of an experiment described in (a) were pooled and analysed with anti-USP7, FLAG (USP17), V5 (USP22) and Strep (USP44) antibodies. Expression of USP9X in conjunction with experiments shown in figure 4c was confirmed with an anti-FLAG antibody.

Supplementary Figure S5

Direct interaction between USP17 and ELK-1 *in vitro*

a, **b**) Recombinant GST-USP17, GST, ELK-1 and ERK2 proteins expressed in E coli, showing relative amounts used in the *in vitro* interaction assays. Arrowheads indicate full length GST-USP17 fusions (a) and ELK-1 proteins (b).

c) Nuclear (N) and cytosolic (C) fractions were prepared from HEK293T cells transfected with vectors for HA.ELK-1 and analysed by SDS-PAGE (5-20%) and immunoblotting with antibodies indicated.

Supplementary Figure S6

Mitogen induction of *CFOS* in HeLa cells

HeLa cells were treated with TPA and MEK inhibitor U0126 as indicated. RNA was isolated and analysed by qRT-PCR for *CFOS* mRNA (n=3).

Supplementary Figure S7

USP17 depletion and cell proliferation

a) HeLa cells were transfected with vectors for shUSP17#1, shUSP17#2 or vector control (pSUPER) as indicated. One day post transfection cells were seeded into 96-well plates and proliferation was assessed every 24h by MTT assay. Data are averages of three independent experiments in which each point is the average from 4 measurements. • = control v. shUSP17#1; * = control v. shUSP17#2.

b) HEK293T cells were transfected with vectors for USP17 sh#1, shELK-1 or vector control (pSUPER) as indicated. One day post transfection cells were counted and re-seeded into 24-well plates and proliferation was assessed after 4 days by automated cell counting. Data are averages of four independent experiments with control normalised to 1.
