# Supplementary figures and images for: De-ubiquitination of ELK-1 by USP17 potentiates mitogenic gene expression and cell proliferation
Source: Nucleic Acids Res. 2019 Mar 11;47(9):4495–508. doi: 10.1093/nar/gkz166 (PMC6511843; doi:10.1093/nar/gkz166)

SUPP. FIGURE S1

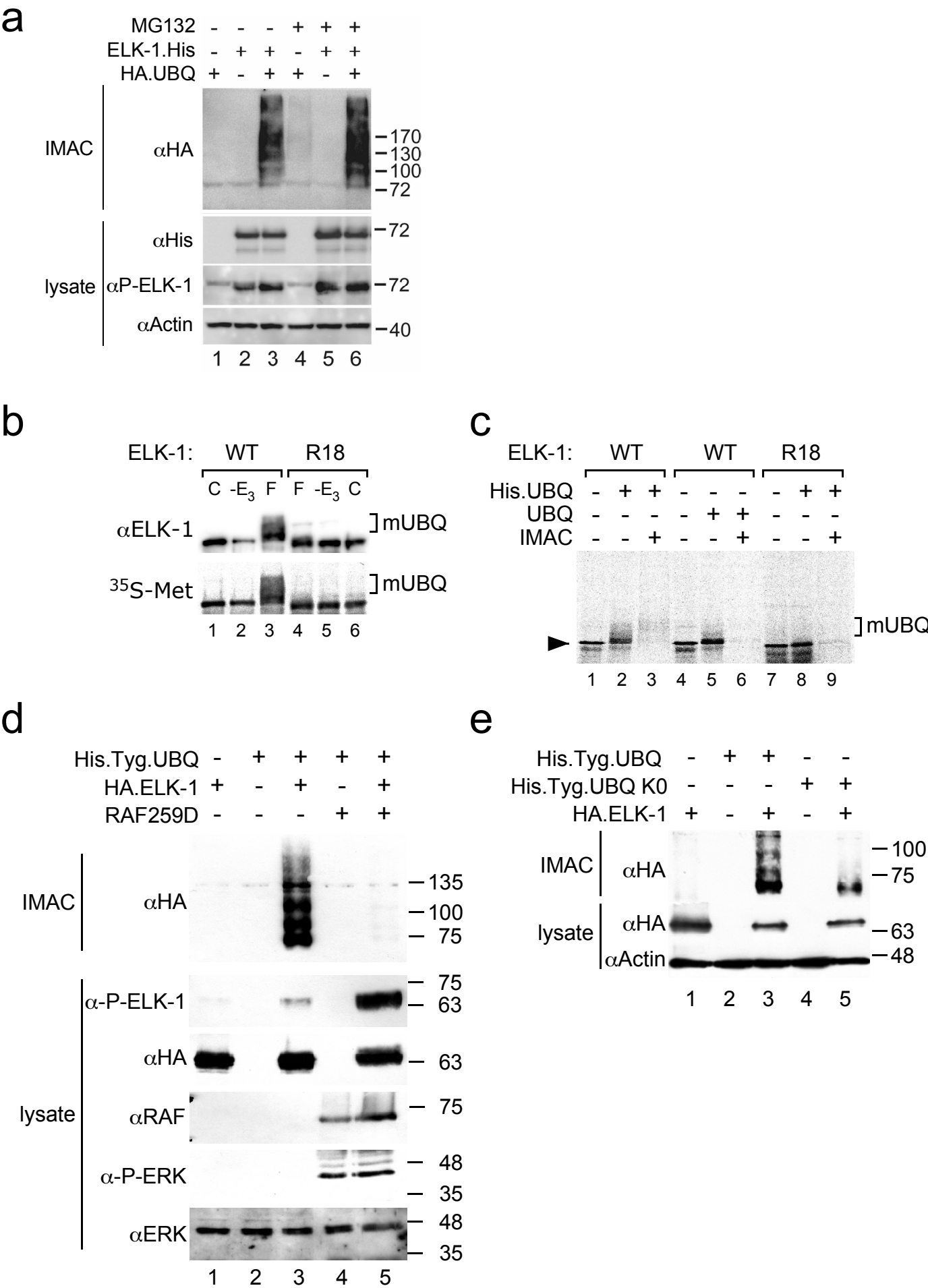

Supplement: Supplementary Data [file gkz166_supplemental_files.zip › ELK MUBI SUPP F1ae JAN.pdf]

a

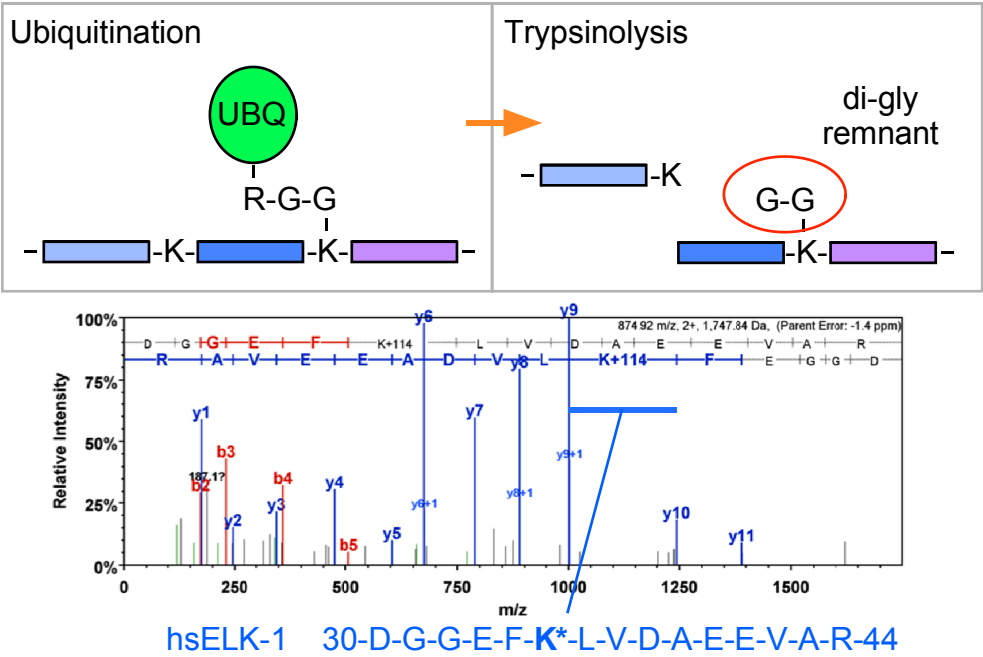

b

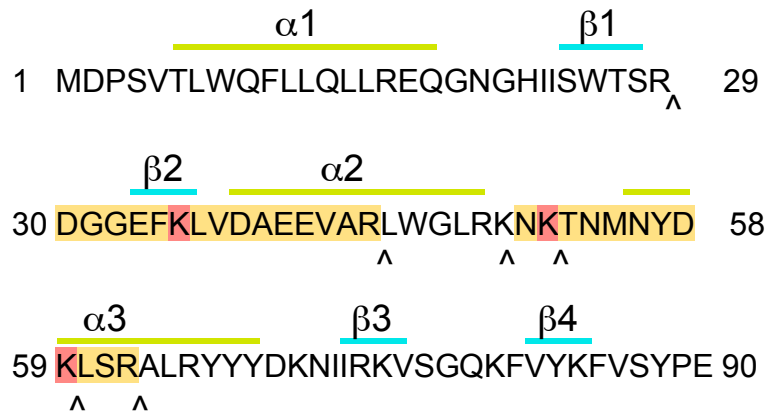

Supplement: Supplementary Data [file gkz166_supplemental_files.zip › ELK MUBI SUPP F2ab JAN.pdf]

SUPP. FIGURE S3

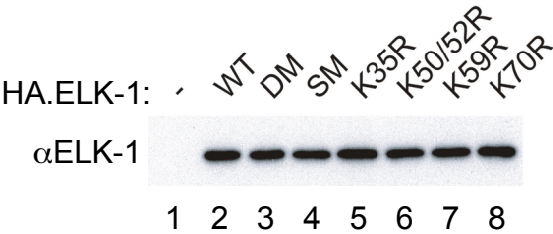

Supplement: Supplementary Data [file gkz166_supplemental_files.zip › ELK MUBI SUPP F3 JAN.pdf]

a

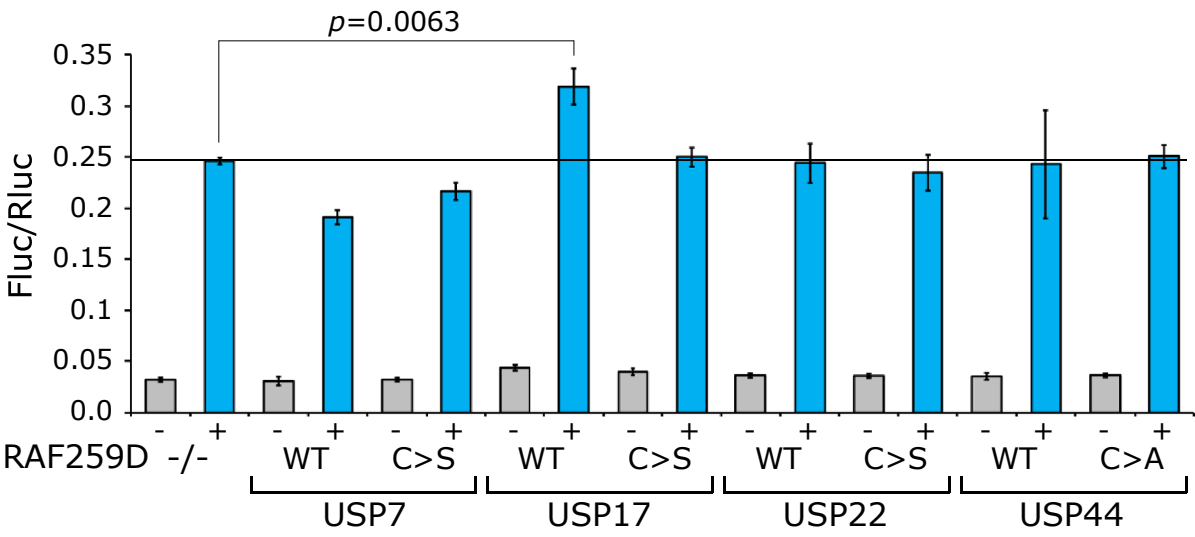

b

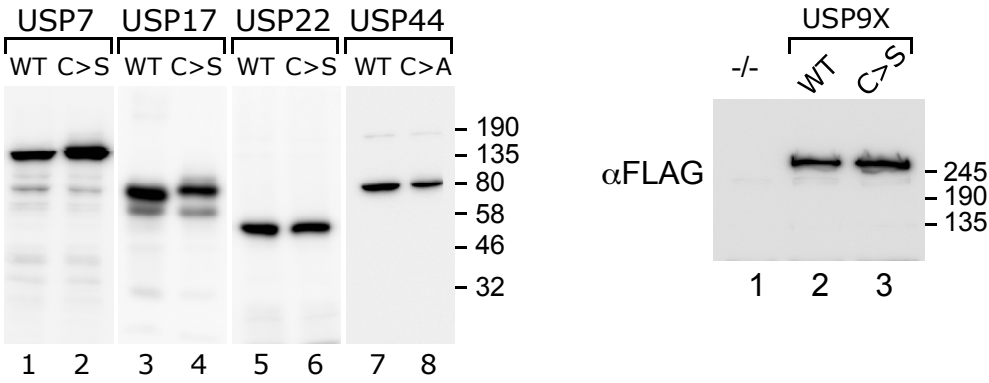

Supplement: Supplementary Data [file gkz166_supplemental_files.zip › ELK MUBI SUPP F4ab JAN.pdf]

a

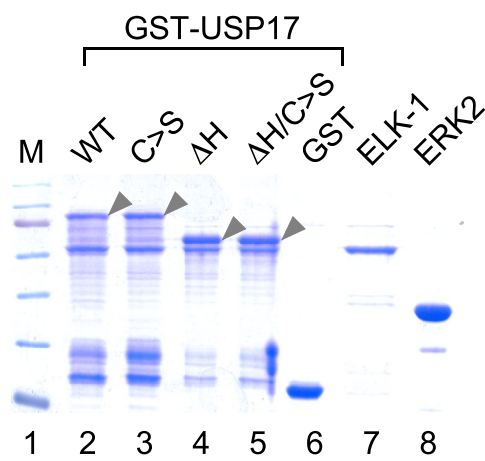

b

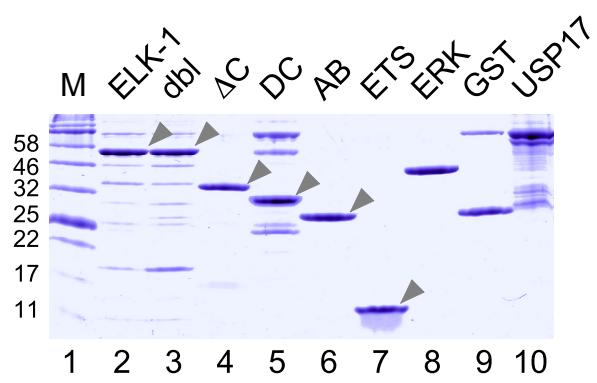

c

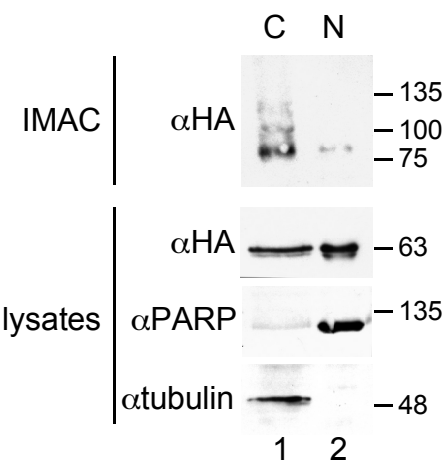

Supplement: Supplementary Data [file gkz166_supplemental_files.zip › ELK MUBI SUPP F5ac JAN.pdf]

SUPP FIGURE 6

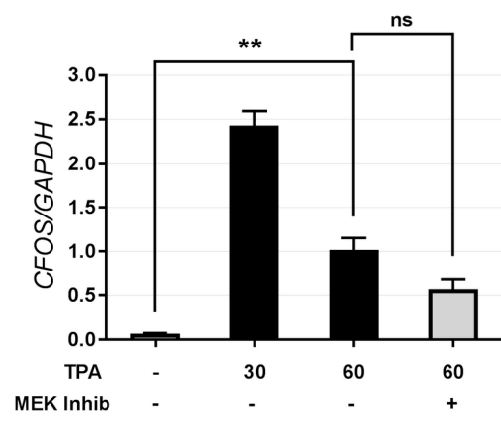

Supplement: Supplementary Data [file gkz166_supplemental_files.zip › ELK MUBI SUPP F6 JAN.pdf]

SUPP. FIGURE S7

a

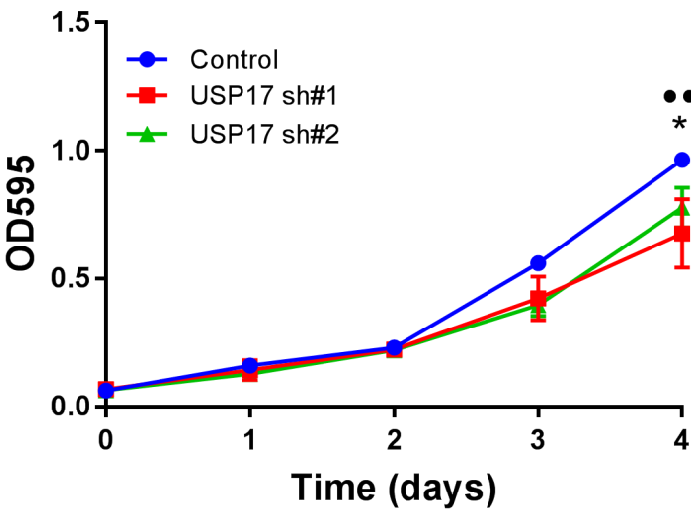

b

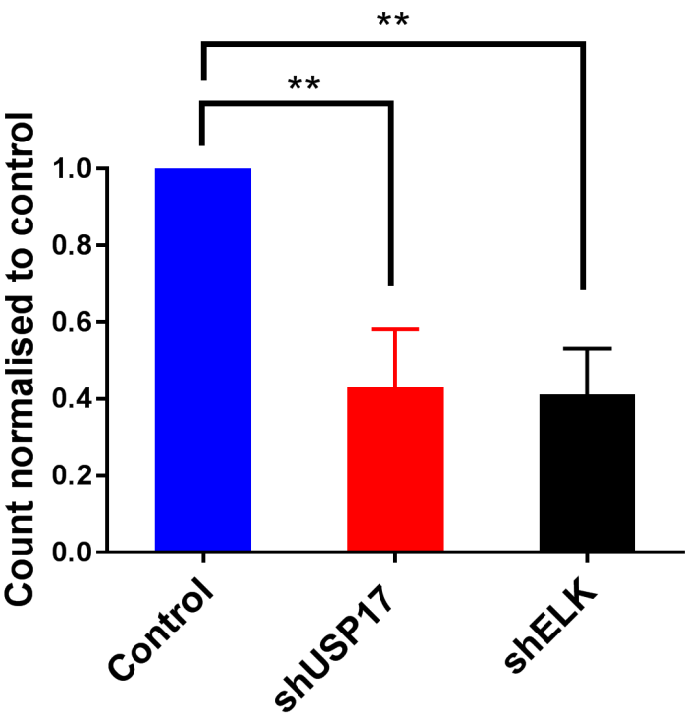

Supplement: Supplementary Data [file gkz166_supplemental_files.zip › ELK MUBI SUPP F7 JAN.pdf]
